# Postoperative intensive care allocation and mortality in high-risk surgical patients: evidence from a low- and middle-income country cohort

**DOI:** 10.1016/j.bjane.2024.844517

**Published:** 2024-05-23

**Authors:** Adriene Stahlschmidt, Sávio Cavalcante Passos, Guilherme Roloff Cardoso, Gabriela Jungblut Schuh, Paulo Corrêa da Silva Neto, Stela Maris de Jezus Castro, Luciana Cadore Stefani

**Affiliations:** a Universidade Federal do Rio Grande do Sul (UFRGS), Faculdade de Medicina, Programa de Pós-Graduação em Ciências Médicas, Porto Alegre, RS, Brazil; b Hospital de Clínicas de Porto Alegre (HCPA), Porto Alegre, RS, Brazil; c Universidade Federal do Rio Grande do Sul (UFRGS), Departamento de Estatística, Porto Alegre, RS, Brazil; d Universidade Federal do Rio Grande do Sul (UFRGS), Faculdade de Medicina, Departamento de Cirurgia, Porto Alegre, RS, Brazil

**Keywords:** Surgical procedures, Operative, Risk factors, Surgical intensive care, Resources allocation, In-hospital mortality, Postoperative Complications/prevention & control

## Abstract

**Background:**

The escalation of surgeries for high-risk patients in Low- and Middle-Income Countries (LMICs) lacks evidence on the positive impact of Intensive Care Unit (ICU) admission and lacks universal criteria for allocation. This study explores the link between postoperative ICU allocation and mortality in high-risk patients within a LMIC. Additionally, it assesses the Ex-Care risk model's utility in guiding postoperative allocation decisions.

**Methods:**

A secondary analysis was conducted in a cohort of high-risk surgical patients from a 800-bed university-affiliated teaching hospital in Southern Brazil (July 2017 to January 2020). Inclusion criteria encompassed 1431 inpatients with Ex-Care Model-assessed all-cause postoperative 30-day mortality risk exceeding 5%. The study compared 30-day mortality outcomes between those allocated to the ICU and the Postanesthetic Care Unit (PACU). Outcomes were also assessed based on Ex-Care risk model classes.

**Results:**

Among 1431 high-risk patients, 250 (17.47%) were directed to the ICU, resulting in 28% in-hospital 30-day mortality, compared to 8.9% in the PACU. However, ICU allocation showed no independent effect on mortality (RR = 0.91; 95% CI 0.68‒1.20). Patients in the highest Ex-Care risk class (Class IV) exhibited a substantial association with mortality (RR = 2.11; 95% CI 1.54–2.90) and were more frequently admitted to the ICU (23.3% vs. 13.1%).

**Conclusion:**

Patients in the highest Ex-Care risk class and those with complications faced elevated mortality risk, irrespective of allocation. Addressing the unmet need for adaptable postoperative care for high-risk patients outside the ICU is crucial in LMICs. Further research is essential to refine criteria and elucidate the utility of risk assessment tools like the Ex-Care model in assisting allocation decisions.

## Introduction

The global demand for surgical procedures is on the rise, surpassing 310 million annually,^1^^,^^2^ particularly in countries with limited healthcare spending (< US$ 40/per capita).^1^ A staggering 4.8 billion people lack access to safe surgical treatments, resulting in a shortfall of at least 143 million surgical procedures yearly, mainly in Low- and Middle-Income Countries (LMIC).^3^ This surge in demand, coupled with the increasing severity of patients, strains healthcare systems, especially in high-complexity settings like Intensive care Units (ICU).

High-risk surgical patients, facing a 5–25% mortality and frequent complications,^4^ often need routine ICU admissions. However, ICU beds are costly, have limited resources, and lack clear criteria for admission leading to potential disparities in access.^2^^,^^5^^,^^6^ In LMICs where resources are scant, this issue becomes critical. In Brazil alone, with 4 million annual surgical procedures and mortality rates ranging from 1.7% to 2.8%,^7^ the high-risk population constitutes 10–12% of patients.^8^^,^^9^ ICU data in Brazil reveal mortality rates of 9.6–16.7% within 30 days and complication rates of 30–38%.^10^

Globally, less than 15% of high-risk surgical patients are admitted to the ICU due to the absence of clear admission criteria.^4^ In Brazil, multicentric studies highlight low rates of ICU admissions after noncardiac surgeries (3.5%) and insufficient standardization regarding indications. In response to these challenges, we developed the Ex-Care risk model to identify high-risk surgical patients,^8^ categorizing them as high-risk (5–10% likelihood of death) or very high-risk (>10% probability of death). A care bundle for these patients was successfully implemented in surgical wards,^11^ with a particular interest in the outcomes of high-risk patients electively admitted to ICU.

The primary objective of this study is to explore the association between postoperative ICU allocation and in-hospital postoperative mortality in a cohort of high-risk surgical patients in an LMIC setting. The secondary aim is to assess the association between Ex-Care risk classes, ICU admissions, and mortality. The hypothesis posits that postoperative ICU allocation influences in-hospital postoperative mortality among high-risk surgical patients in LMICs. Furthermore, it suggests an association between Ex-Care risk classes, ICU admissions, and mortality.

## Methods

### Study design and setting

This is a secondary analysis of a follow-up study of high-risk surgical patients from a single-center, 800-bed quaternary hospital in southern Brazil (Hospital de Clínicas de Porto Alegre – HCPA). The protocol was approved by the National Research Ethics Committee (CAAE 0444.8018.8.0000.5327) and registered in Clinical Trials (identifier: NCT04187664). The study was approved by the local Institutional Review Board, which granted informed consent clearance. Our report was based on the STROBE (STrengthening the Reporting of OBservational studies in Epidemiology) statement (Supplementary material S1 Table).^12^

### Study population and data source

The cohort comprised consecutive high-risk adults that underwent surgery between July 2017 and January 2020 and were referred either to the ICU or PACU immediately after surgery. High surgical risk, defined as probability of death ≥5%, was determined using the Ex-Care model^8^ (Supplementary material S2 Table). We excluded patients aged less than 16 years, and those who underwent outpatient, diagnostic and cardiac procedures, transplants, and procedures under local anesthesia.

Data on the surgical procedure, comorbidities, complications, and outcomes were obtained from electronic medical records and corrected for missing values using complete case analysis. The researchers were physicians trained to find information of preoperative comorbidities, surgery/anesthesia, postoperative complications, and deaths (the detailed variables collected are outlined in the section below). We defined pre-existing medical conditions as those fulfilling at least one of the following: appeared in medical records; registered in preanesthetic documents; confirmed by laboratory results.

### Outcomes definition

The primary outcome was in-hospital postoperative mortality, censored at 30 days after surgery if the patient was still alive and in hospital. Secondary outcome was mortality in patients allocated at an ICU within 30 days after surgery, based on their respective Ex-Care risk model classes.

### Exposure

The exposure of interest was immediate postoperative allocation at either ICU or PACU. The ICU group comprised patients who were directly transferred to an ICU as determined by the surgical/anesthetic team and were not in the PACU at any time. Criteria for direct postoperative ICU admission vary among surgical specialties, surgical severity, and ICU availability. Organ dysfunction or instabilities are the main prioritization criteria for ICU allocation in our institution.

The PACU group comprised high-risk patient's ineligible for ICU or due to ICU bed unavailability. The PACU emphasizes short-stay recovery and is staffed by consultant anesthesiologists and nurses to provide 24-hour care. It focuses on continuous monitoring, pain management, hemodynamic optimization, and immediate postoperative complications. After meeting discharge criteria (Supplementary material S3 Table), patients transition to surgical wards. Admission, staffing, and physical structure criteria for ICU/PACU/standard wards remained consistent throughout the study.

To adjust for different sites of postoperative allocation, high-risk patients were classified using the Ex-Care model, based on death probability within 30 days (Class III: 5.0–9.9%, Class IV ≥ 10%).^8^ Developed and validated in the same hospital, the model, encompassing age, ASA-PS (American Society of Anesthesiologists physical status), surgical severity, and nature, demonstrated high discriminative power for postoperative in-hospital death. A non-proprietary smartphone application for the Ex-Care model is available on mobile platforms.

### Variables

In the analysis, variables included age, gender, ASA-PS,^13^ surgical severity^14^ and nature (elective or urgent). Surgical specialties were categorized into general (upper abdomen, lower gastrointestinal and hepatobiliary), vascular, orthopedics, urology, thoracic, neurosurgery and others (breast, head and neck, gynecology, and plastics).

Major complications within 7 days were: previous diagnosis of ventilatory support, postoperative vasopressor usage, acute kidney injury, transfusion, myocardial infarction, stroke, arrhythmia, thromboembolic event, delirium, wound dehiscence, sepsis, abdominal complication, or surgical reintervention within 7 days. Data collection followed definitions from the European Perioperative Clinical Outcome (EPCO),^15^ World Health Organization (WHO), Third International Consensus Definitions for Sepsis and Septic Shock (Sepsis-3), and POMS questionnaire domains^16^^,^^17^ (Supplementary material S4 Table).

Process outcomes were additionally assessed, including unplanned readmissions for patients previously assigned to the ICU or unplanned ICU admissions for those primarily designated to PACU, calls to the Rapid Response Team (RRT) within 7 days, and length of hospital stay. Hospital readmissions and unplanned surgical reinterventions within 30 days were also recorded.

### Statistical analysis

The baseline characteristics of the two cohorts (ICU and PACU) were described in terms of continuous and categorical variables. The crude rates of death, complications and process measures were presented according to the postoperative allocation and Ex-Care risk classes.

For the primary analysis, we conducted a complete case analysis, excluding patients with missing data. We examined the independent association between in-hospital 30-day mortality and postoperative ICU allocation using Poisson regression with robust error variance, capable of directly estimating relative risks and avoiding the risk of overestimations from odds ratios.

To control for potential confounding factors, we built successive multilevel models adjusting for a group of variables based on a conceptual framework that describes the relationship between risk-factors.^18^ For all models, forced simultaneous entry (all candidate variables remained in the model) was used rather than automated stepwise selection. The blocks of covariates were: first, preoperative risk class defined by Ex-Care model; second, surgical specialties (general surgery as the reference); third, postoperative complications (transfusion, cardiovascular, abdominal, sepsis, delirium, and acute kidney injury); fourth, postoperative instability (need of ventilatory or hemodynamic support). A power analysis was not conducted for this exploratory study, which relied on a convenience sample.

The significance level for all analyzes was 5%. Data were analyzed using the Statistical Analysis System (SAS Studio® 9.4. Cary. NC. USA) and R (version 3.5.1).

## Results

### Patient selection and postoperative allocation

During the study period, a total of 30,419 surgeries were performed. After excluding 15,983 cases (reoperations, transplants, diagnostic, cardiac and ambulatory procedures), 14,436 procedures were conducted at the main surgical unit, with 1,208 (8.36%) directly admitted to ICU. Among the 1,558 (10.79%) high-risk patients, 1,431 were eligible after excluding 127 patients who were preoperatively in the ICU. Postoperatively, 250 (17.47%) high-risk patients were referred to the ICU.

**Figure 1:**
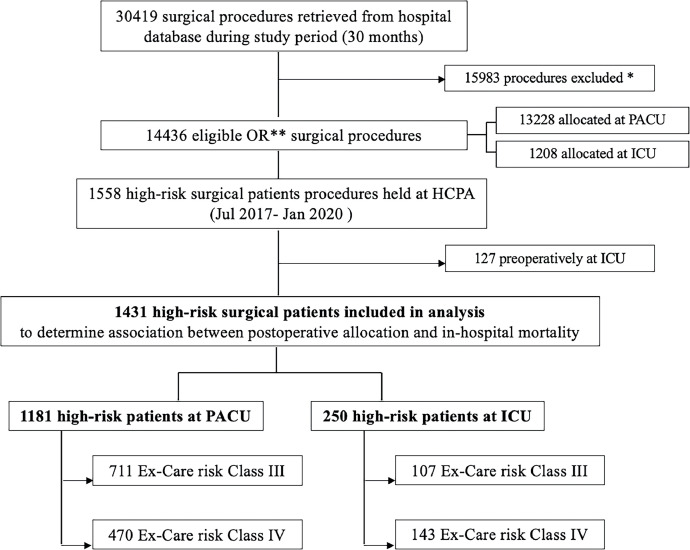
Flow chart of study cohort selection. OR, Operating Room; ICU, Intensive Care Unit; PACU, Postanesthetic Care Unit; HCPA, Hospital de Clínicas de Porto Alegre. * Reoperations, transplants, diagnostic, cardiac and ambulatory procedures. ** Procedures held at the main surgical unit.

Regarding risk class, 23.3% (143/613) of the very high-risk group (Ex-Care Class IV) and 13.0% (107/818) of the high-risk group (Ex-Care Class III) were allocated to the ICU. Patient inclusion is depicted in Figure 1.

### High-risk patient characteristics

The mean (SD) age of patients allocated at ICU was 65.72 (12.61) and 68.42 (11.3) years for the PACU group. Among patients directly admitted to the ICU in the postoperative period, there was a higher proportion of ASA-PS ≥ IV (28.0% vs. 10.2% at PACU, *p* < 0.01), and of those with predicted mortality ≥10% (57.2% vs. 39.8% at PACU, *p* < 0.01). Baseline characteristics of the study population are shown in Table 1.

**Table 1:** Clinical characteristics stratified by postoperative allocation group.

	Missing data, n (%)				*p-*value
	PACU (n = 1181)	ICU (n = 250)	PACU (n = 1181)	ICU (n = 250)	
**Demographic**					
Age (yr)	68.42 (11.03)	65.72 (12.61)	‒	‒	<0.01
Male	671 (56.8)	122 (48.8)	‒	‒	0.31
**Composite risk scales**					
ASA-PS					<0.01
II	25 (2.1)	2 (0.8)	‒	‒	
III	1035 (87.6)	178 (71.2)			
IV	117 (9.9)	58 (23.2)			
V	4 (0.3)	12 (4.8)			
Risk of death (Ex-Care model)^a^					<0.01
Predicted mortality 5.0–9.9%	711 (60.2)	107 (42.8)	‒	‒	
Predicted mortality ≥ 10%	470 (39.8)	143 (57.2)			
**Operative**					
Procedure type					<0.01
Abdominal	411 (34.8)	103 (41.2)	‒	‒	
Vascular	287 (24.3)	40 (16.0)			
Orthopedic	103 (8.7)	9 (3.6)			
Urologic	141 (11.9)	23 (9.2)			
Thoracic	113 (9.6)	8 (3.2)			
Neurosurgical	40 (3.4)	60 (24.0)			
Other	86 (7.3)	7 (2.8)			
Surgical severity					<0.01
Minor	147 (12.4)	14 (5.6)	‒	‒	
Intermediate	254 (21.5)	30 (12)			
Major	780 (66.0)	206 (82.4)			
Urgent surgery	556 (47.1)	124 (49.6)	‒	‒	0.46
**Comorbidities**					
Coronary artery disease	204 (17.3)	39 (15.6)	‒	‒	0.52
Heart failure	114 (9.7)	25 (10.0)	‒	‒	0.86
COPD	170 (14.4)	46 (18.4)	‒	‒	0.10
Cancer	500 (42.3%)	125 (50.0)	‒	‒	0.02
Insulin-dependent DM	176 (14.9%)	32 (12.8)	1	‒	0.38
Cerebrovascular disease	181 (15.3%)	39 (15.6)	‒	‒	0.91
Sepsis	87 (7.4%)	52 (20.8)	‒	1	<0.01
Anemia^b^	764 (64.8%)	148 (59.7)	2	‒	0.12
Dialysis	65 (5.7%)	65 (5.7)	50	8	0.45
Acute kidney injury	150 (13.0%)	42 (16.5%)	31	1	0.15
Chronic renal failure	344 (30.0%)	68 (27.3%)	33	2	0.40

All characteristics have no missing data unless otherwise stated. Data are presented as mean (±SD), median (IQR) or absolute values (%). Patient ages and hemoglobin were compared with Student's *t*-test; serum creatinine, eGFR, surgical duration and length of stay were compared with Mann-Whitney test; all other comparisons were performed with two-tailed Chi-Square test.

a Ex-Care risk model was determined using calculator available online.

b Anemia was defined as Hemoglobin (Hb) < 13 g.dL^−1^ for men and < 12 g.dL^−1^ for women. PACU, Postanesthetic Care Unit; ICU, Intensive Care Unit; ASA-PS, American Society of Anesthesiologists Physical Status; COPD, Chronic Obstructive Pulmonary Disease; DM, Diabetes Mellitus; PO, Postoperative.

Regarding surgical aspects, the nature of the procedures (elective or urgent) was not different between groups while major surgeries were more frequent in the ICU group (82.4% vs. 66.0% at PACU, *p* < 0.01). The most prevalent surgeries were abdominal, vascular, urological, and orthopedic. Neurosurgical patients were more frequently admitted to the ICU (24.0% vs. 3.4% at PACU, *p* < 0.01). As for comorbidities, there was a higher incidence of cancer and preoperative sepsis in those admitted to the ICU (Table 1).

### Incidence of complications

Major complications occurred in 79.6% of patients allocated to the ICU versus 49.1% of those referred to PACU (*p* < 0.01). Patients allocated to the ICU had a higher incidence of bleeding and transfusion, ventilatory support (51.6% vs. 7.3% at PACU, *p* < 0.01), hemodynamic support (55.6% vs. 0.4% at PACU, *p* < 0.01), acute kidney injury, arrhythmia, delirium, abdominal complications, and sepsis. We observed no differences in hospital readmission between groups (13.4% vs. 12.4% in PACU group, *p* = 0.67). More surgical reinterventions occurred in the PACU group (11.8% vs. 7.2% in ICU, *p* < 0.03). Table 2 describes the outcomes.

**Table 2:** Process measures, postoperative complications, and mortality by allocation.

	PACU (n = 1181)	ICU (n = 250)	*p-*value
**Process Measures**			
Unplanned ICU admission (30 days)	89 (7.5%)	19 (7.6%)	0.96
Mortality (30 days)	41 (46.0%)	8 (42.1%)	0.87
RRT Calls (7 days)	199 (16.9%)	27 (10.8 %)	0.01
PO Length of stay (days); median (IQR)	7 (4–11)	10 (6–17)	<0.01
**Complications (7 days)**			
Surgical bleeding > 500 mL	144 (12.2%)	71 (28.8%)	<0.01
Ventilatory support	86 (7.3%)	129 (51.6%)	<0.01
Hemodynamic support	123 (10.4%)	139 (55.6%)	<0.01
Acute kidney injury	176 (14.9%)	58 (23.2%)	0.01
Transfusion	161 (13.6%)	66 (26.4%)	<0.01
Myocardial infarction	11 (0.9%)	5 (2.0%)	0.30
Stroke	10 (0.8%)	4 (1.6%)	0.27
Arrhythmia	49(4.1%)	27 (10.8%)	<0.01
Thromboembolic event	102 (8.6%)	29(11.6%)	0.14
Delirium	83 (7%)	32 (12.8%)	0.02
Wound dehiscence	18 (1.5%)	10 (0.8%)	0.37
Abdominal	171 (14.5%)	63 (25.2%)	<0.01
Sepsis	91 (7.7%)	63 (25.2%)	<0.01
**Hospital readmission (30 days)**	158(13.4%)	31 (12.4%)	0.67
**Surgical reintervention (30 days)**	139 (11.8%)	18 (7.2%)	<0.03
**Major complication**^a^	580 (49.1%)	199 (79.6%)	<0.01
**30-day mortality**	105 (8.9%)	70 (28.0%)	<0.01

Data are presented as median (IQR) or absolute values (%). PACU, Postanesthetic Care Unit; ICU, Intensive Care Unit; RRT, Rapid Response Team; PO, Postoperative.

a Major complication: previous diagnosis of ventilatory support, postoperative vasopressor usage, acute kidney injury, transfusion, myocardial infarction, stroke, arrhythmia, thromboembolic event, delirium, wound dehiscence, sepsis, abdominal complication, or surgical reintervention within 7 days.

Data regarding process outcomes showed no difference in unplanned admission or readmission to ICU (7.6% vs. 7.5% in PACU group, *p* = 0.96). Furthermore, for those who experienced unplanned ICU admission, mortality exceeded 40% in both groups (46.0% in PACU group vs. 42.1% in ICU group, *p* = 0.87). Postoperative length of stay was significantly longer in the ICU group, with a median of 10 (6–17) vs. 7 (4–11) days for those allocated at PACU (Table 2).

### Association between mortality and postoperative allocation

Amongst 1431 high-risk surgical patients included in the analysis we identified 12.2% overall mortality. In the group allocated at the ICU, 70/250 patients (28.0%) died within 30 days vs. 105/1181 (8.9%) allocated at the PACU (Table 2). Before adjustment, the RR for death was 3.19 (95% CI 2.43–4.20) for ICU in relation to the PACU group. We adjusted risk for confounding factors included in successive models that considered preoperative risk class, surgical specialty, postoperative complications, and instabilities (need for hemodynamic and/or ventilatory support). After risk adjustment, there was no independent effect of ICU allocation in the immediate postoperative period on mortality (RR = 0.91; 95% CI 0.68–1.20) (Table 3).

**Table 3:** Unadjusted and adjusted association between ICU allocation and in-hospital death within 30 days in 1431 patients – Poisson regression model.

Baseline Model	RR (95% CI)	*p-*value
ICU allocation vs. PACU	3.19 (2.43–4.2)	<0.01
**Model 1: preoperative risk**		
ICU allocation vs. PACU	2.41 (1.78–3.26)	<0.01
Ex-Care model^a^		
Predicted mortality 5.0–9.9%	1 (REF)	
Predicted mortality ≥ 10%	4.26 (3.12–5.81)	<0.01
**Model 2: preoperative risk + specialty**		
ICU allocation vs. PACU	2.50 (1.82–3.43)	<0.01
Ex-Care model risk class		
III (predicted mortality 5.0–9.9%)	1 (REF)	
IV (predicted mortality ≥ 10%)	3.74 (2.68–5.21)	<0.01
Surgical Specialty		
General	1 (REF)	
Vascular	0.69 (0.46–1.04)	0.08
Orthopedics	0.77 (0.40–1.46)	0.42
Urology	0.79 (0.48–1.30)	0.36
Thoracic	0.78 (0.43–1.42)	0.42
Neurosurgery	0.49 (0.29–0.82)	<0.01
Other	0.37 (1.14–0.99)	<0.05
**Model 3: preoperative risk + specialty + PO complications**		
ICU allocation vs. PACU	1.42 (1.04–1.95)	0.02
Ex-Care model risk class		
III (predicted mortality 5.0–9.9%)	1 (REF)	
IV (predicted mortality ≥ 10%)	2.27 (1.60–3.23)	<0.01
Surgical Specialty		
General	1 (REF)	
Vascular	1.11 (0.73–1.67)	0.61
Orthopedics	1.11 (0.64–1.93)	0.68
Urology	0.94 (0.50–1.52)	0.80
Thoracic	1.26 (0.70–2.28)	0.43
Neurosurgery	1.49 (0.86–2.15)	0.15
Other	0.61 (0.23–1.60)	0.31
PO complications		
PO transfusion	1.20 (0.87–1.66)	0.25
Cardiovascular	0.98 (0.63–1.54)	0.95
Abdominal	1.50 (1.04–2.14)	0.02
Sepsis	4.14 (2.85–6.02)	<0.01
Delirium	1.20 (0.83–1.74)	0.31
Acute kidney injury	2.28 (1.69–3.07)	<0.01
**Final Model**^b^**: preoperative risk + specialty + PO complications + PO instability**		
**ICU allocation vs. PACU**	**0.91 (0.68–1.20)**	**0.52**
Ex-Care model risk class		
III (predicted mortality 5.0–9.9%)	1 (REF)	
IV (predicted mortality ≥ 10%)	2.11 (1.54–2.90)	<0.01
Surgical Specialty		
General	1 (REF)	
Vascular	1.08 (0.68–1.49)	0.96
Orthopedics	1.26 (0.72–2.19)	0.41
Urology	0.92 (0.58–1.44)	0.71
Thoracic	1.10 (0.61–1.98)	0.74
Neurosurgery	1.59 (0.95–2.68)	0.07
Other	0.65 (0.25–1.68)	0.37
PO complications		
PO transfusion	1.06 (0.79–1.42)	0.68
Cardiovascular^b^	0.98 (0.63–1.50)	0.92
Abdominal	1.47 (1.06–2.05)	0.02
Sepsis	2.63 (1.84–3.77)	<0.01
Delirium	1.20 (0.85–1.68)	0.28
Acute kidney injury	1.87 (1.41–2.49)	<0.01
PO instability^c^	3.91 (2.63–5.80)	<0.01

RR, Relative Risk; CI, Confidence Interval; ICU, Intensive Care Unit; PACU, Postanesthetic Care Unit; REF, Reference Category; PO, Postoperative.

a Ex-Care risk model considers age, ASA-PS, nature (urgent or elective) or severity of surgery (major vs. non-major).

b Including myocardial infarction, arrhythmias, and stroke.

c PO instability was defined as the need of ventilatory or hemodynamic support.

In this analysis, the independent factors associated with 30-day postoperative mortality were Ex-Care Class IV (RR = 2.11; 95% CI 1.54–2.90), postoperative abdominal complications (RR 1.47; 95% CI 1.06–2.05), sepsis (RR 2.63; 95% CI 1.1.84–3.77) and acute kidney injury (RR = 1.87; 95% CI 1.41–2.49). An association of mortality with postoperative hemodynamic and/or ventilatory instabilities was also demonstrated (RR = 3.91; 95% CI 2.63–5.80). The adjusted RRs associated with mortality for variables included in the multivariable models are in Table 3.

### Association between Ex-Care risk classes and mortality

To identify whether very high-risk patients (predicted mortality ≥ 10%) could benefit from allocation to intensive care, we analyzed two subgroups of patients regarding their Ex-Care risk classes (Class III and IV). We explored inpatient 30-day mortality and its relationship with surgical aspects, postoperative complications, and allocation.

**Figure 2:**
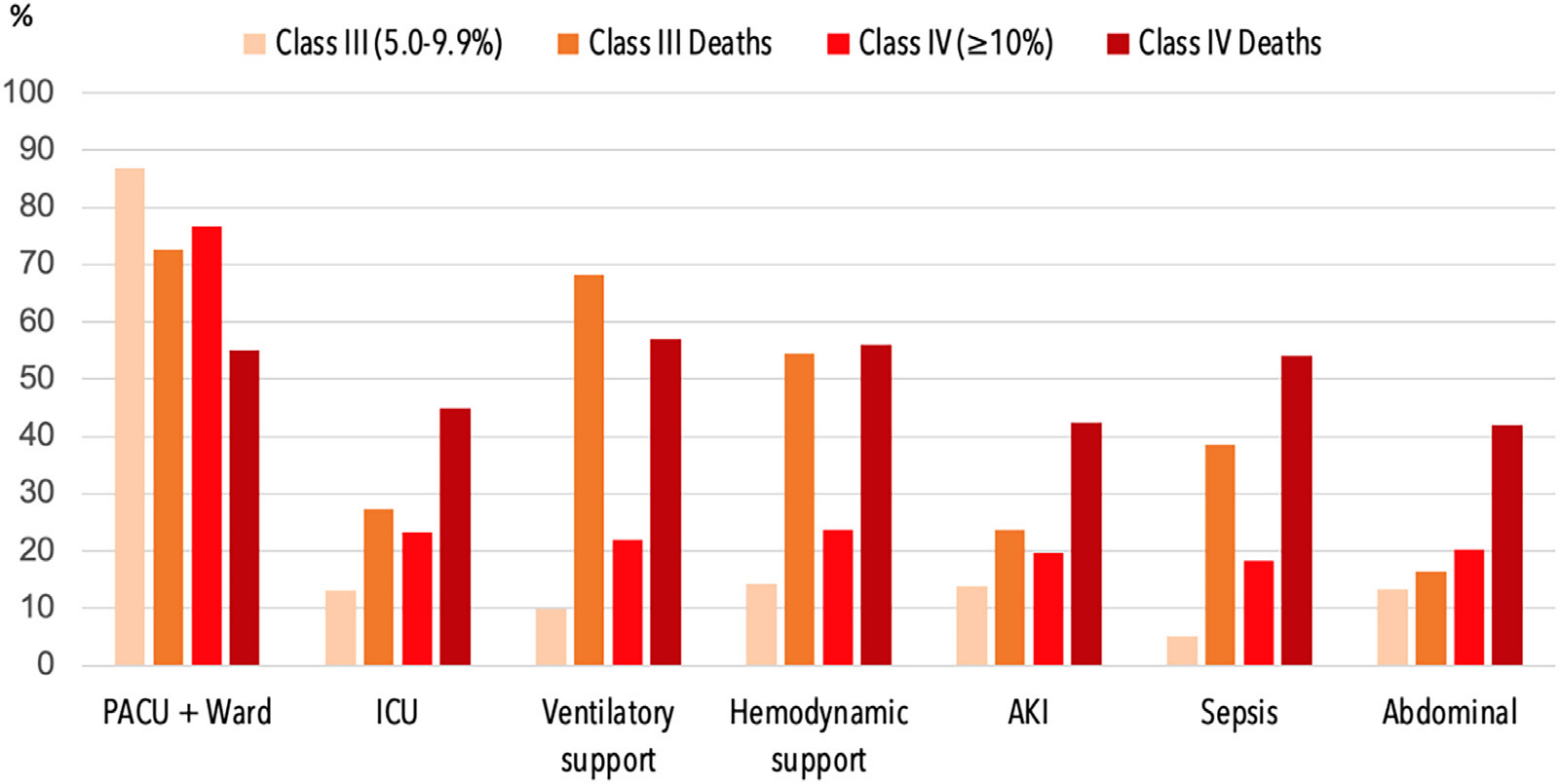
Outcomes according to each risk class of Ex-Care model in high-risk surgical patients. PACU, Postanesthetic Care Unit; ICU, Intensive Care Unit; AKI, Acute Kidney Injury.

The subgroup of Class IV patients confirmed predictions with substantially higher mortality rates (20.71% vs. 5.37% at Class III). Class IV patients were more frequent at general surgery procedures (44.0%) and reinterventions (12.6%). Major postoperative complications occurred in above 90% of high-risk patients who died in contrast with rates of 43.0% at risk Class III and 63.3% at risk Class IV. The most frequent complications and their rates by subgroup are presented at Figure 2. The group of Class IV patients was more frequently admitted to ICU (23.3% vs. 13.1% of Class III). The rates of ICU admission were higher also for Class IV patients who died within 30 days (44.9% vs. 27.3% of Class III) (Fig. 2 and Table 4). Elsewhere, at the final Poisson regression model, the very-high risk group demonstrated to be strongly associated with mortality (RR = 2.11; 95% CI 1.54–2.90).

**Table 4:** Outcomes according to each risk class of Ex-Care model in high-risk surgical patients.

	Class III (mortality 5.0%‒9.9%)		Class IV (mortality ≥ 10%)	
	Total (n = 818)	Deaths (n = 44)	Total (n = 613)	Deaths (n = 127)
**Operative**				
Surgical Specialty				
General	245 (30.0)	14 (5.7)	269 (44.0)	74 (27.5)
Vascular	195 (23.8)	10 (5.1)	132 (21.5)	19 (14.4)
Orthopedics	63 (7.7)	5 (7.9)	49 (8.0)	5 (10.2)
Urology	98 (12.0)	5 (5.1)	66 (10.8)	12 (18.2)
Thoracic	90 (11.0)	6 (6.7)	31 (5.1)	5 (16.1)
Neurosurgery	60 (7.3)	2 (3.3)	40 (6.5)	10 (25.0)
Other	67 (8.2)	2 (3.0)	26 (4.2)	2 (7.7)
Surgical bleeding > 500 mL	145 (17.7)	11 (25.0)	71 (11.6)	14 (11.0)
Reintervention	80 (9.8)	7 (15.9)	77 (12.6)	14 (18.2)
**PO complications**				
Ventilatory support	80 (9.8)	3 (6.8)	135 (22.0)	71 (57.0)
Hemodynamic support	117 (14.3)	24 (54.5)	145 (23.7)	71 (56.0)
Acute kidney injury	114 (13.9)	27 (23.7)	120 (19.6)	51 (42.5)
Thromboembolic event	61 (7.5)	3 (6.8)	70 (11.4)	12 (17.1)
Abdominal	110 (13.4)	18 (16.4)	124 (20.2)	52 (42.0)
Sepsis	42 (5.1)	17 (38.6)	112 (18.3)	69 (54.0)
Major complication^a^	352 (43.0)	41 (93.2)	390 (63.3)	115 (90.5)
**Allocation**				
PACU	711 (86.9)	32 (72.7)	470 (76.6)	70 (55.1)
ICU	107 (13.1)	12 (27.3)	143 (23.3)	57 (44.9)

Data are presented as absolute values (%). PACU, Postanesthetic Care Unit; ICU, Intensive Care Unit; PO, Postoperative.

a Major complication: previous diagnosis of ventilatory support, postoperative vasopressor usage, acute kidney injury, transfusion, myocardial infarction, stroke, arrhythmia, thromboembolic event, delirium, wound dehiscence, sepsis, abdominal complication, or surgical reintervention within 7 days.

## Discussion

The primary objective of our study was to evaluate the outcomes and allocation scenarios for high-risk surgical patients in a Brazilian public hospital setting. Our analysis revealed that 8.3% of all surgical inpatients and only 17.4% of high-risk inpatients had planned ICU admissions. A key finding emerged as we failed to identify evidence supporting the beneficial impact of postoperative critical care on the survival of high-risk surgical patients. Notably, patients in the highest Ex-Care preoperative risk class (≥10%) and those experiencing complications like sepsis, acute kidney injury, or hemodynamic/ventilatory instabilities faced a significantly elevated risk of death, regardless of allocation.

Our finding, indicating that ICU allocation does not correlate with a survival advantage, aligns with findings from previous cohorts.^19^^,^^20^ Additionally, our cohort's ICU admission rates slightly exceeded those from the EuSOS^21^ and from the South-African study SASOS,^22^ possibly due to our institution's nature as a quaternary and regional reference for complex cases. A noteworthy fact is that only 23.1% of all ICU admissions were high-risk patients. More than 70% of high-risk and 55% of very-high-risk patients who died were not immediately allocated to ICU postoperatively. In a similar way, the majority of EuSOS patients who died (73%) were not admitted to ICU at any time, and among those who died after ICU admission, 43% occurred after an uneventful first passage through the unit.^21^ Similarly, the mortality after unplanned ICU admissions in our study exceeded 40%. These findings emphasize a systematic failure in the allocation process of critical care resources, particularly in high-complexity hospitals in LMIC.^22^

The inconsistency in results regarding the potential mortality-reducing effects of ICU allocation and the observed increase in unfavorable outcomes raise questions about routine ICU allocation and the criteria guiding it.^23^ A multicenter study by the International Surgical Outcomes Study group found an association between ICU allocation and increased mortality in high-risk patients (OR = 2.32; 95% CI 1.44–3.74).^2^ Conversely, a UK-based population study reported lowest mortality rates in trusts with a higher number of critical care beds relative to provider size.^24^ This indicates a complex relationship influenced by unadjusted confounding variables, since they are population-based studies using standard databases.

Our study highlights how such confounders considerably impact the final model, and perhaps contributes to identifying the variables that actually lead to high mortality rates besides the place of allocation itself. In fact, the high rate of major complications reported (close to 80% in the ICU group and 50% in the PACU), underscores the significant impact of unstable patients on mortality. On the other hand, as described by Gillies et al, the traditional role of an ICU is to provide organ support, such as invasive ventilation, inotropes/vasopressors, and renal replacement therapy in unstable patients.^25^ The Society of Critical Care Medicine guidelines include as ICU/level 3 and highest priority level (Priority 1) critically ill patients who require organ support and intensive monitoring.^26^

However, few surgical patients require organ support after surgery, even in the high-risk group. What these patients need is prompt treatment of pain, hypothermia, mild cardiorespiratory compromise and fluid imbalance, early mobilization, enteral nutrition, and surveillance for deterioration signs. Surgical teams often refer their patients to ICUs because their staff is used to meeting those needs, but other units can also provide such proactive care. For instance, we identified at our setting a high prevalence of neurosurgical patients admitted to ICU (24.0% vs. 3.4% at PACU), especially due to sensory monitoring demand. We have already shown that adequate surgical ward teams improve patient safety and can reduce the incidence of postoperative complications and mortality.^11^ Therefore, in hospitals that provide excellent ward-based care, the incremental benefit of ICU admissions for stable patients will be reduced.^27^

To address the subjectivity in postoperative allocation decisions and optimize the use of this precious resource, we advocate for better criteria and instruments. Technical basis becomes even more necessary considering the great inequity of ICU beds in the Brazilian Unified Health System (SUS ‒ Sistema Único de Saúde).^28^ Although guidelines suggest not using scoring systems alone to determine the level of care because they are not highly accurate in predicting individual mortality, they can offer guidance when considering physiologic variables.

Our study focused on postoperative allocation in a limited-resource setting, using preoperative risk classes defined by the Ex-Care risk model. Notably, very high-risk patients (Class IV) showed significantly higher rates of intensive care admissions, particularly among those who died. Implementing such risk models into routine clinical practice could enhance decision-making for perioperative teams, map critical bed utilization, and streamline surgical schedules. This finding is corroborated by the Royal College of Surgeons of England's recommendation that all high-risk general surgical patients should be considered for critical care and, as a minimum, patients with an estimated risk of death of ≥ 10%.^29^ Other major studies worldwide, such as the Surgical Risk Preoperative Assessment System (SURPAS)^5^ and the Combined Assessment of Risk Encountered in Surgery (CARES),^30^ have also shown promising results using a surgical risk calculator for accurate prediction of mortality and need for ICU admission.

Among the strengths of our study are the significant number of high-risk patients and using a context-sensitive risk stratification model which allowed comparison for similar patients allocated to different standards of care. Moreover, the adjustment of baseline risk variables and the description of postoperative complications, individually reviewed in medical records, enabled building robust statistical models. Furthermore, our data can be relevant to plan critical care in resource-limited environments.

The study has several limitations. Firstly, being a single-center study restricts external validity. While conducted at a highly representative public hospital, it does not encompass minor district hospitals or private healthcare facilities. Secondly, its observational nature introduces bias and precludes drawing conclusions regarding causality. However, conducting randomized clinical trials to evaluate care processes is challenging. Thirdly, we assessed outcomes solely in the immediate postoperative period, lacking insights into long-term mortality, functional independence, or quality of life. Moreover, the lack of detailed information on illness severity (SAPS, POSSUM, SOFA) restricts the conclusions. Lastly, a power analysis was not conducted for this exploratory study, which relied on a convenience sample.

In conclusion, our study provides valuable insights into the complex decision-making process of postoperative ICU allocation. While we were unable to establish a direct impact of ICU allocation on outcomes, we found indications that ICU stay itself does not significantly affect mortality. Therefore, addressing the unmet need for adaptable postoperative care for high-risk surgical patients outside the ICU is crucial, particularly in LMICs. Further research is essential to refine criteria and elucidate the utility of risk assessment tools such as the Ex-Care model in assisting allocation decisions, especially in resource-limited countries.

## Conflicts of interest

The authors declare no conflicts of interest.

## Appendix Supplementary materials

Image, application 1

## Footnotes

Supplementary material associated with this article can be found in the online version at doi:10.1016/j.bjane.2024.844517.
