# Supplementary material for: Postoperative intensive care allocation and mortality in high-risk surgical patients: evidence from a low- and middle-income country cohort
Source: Braz J Anesthesiol. 2024 May 23;74(4):844517. doi: 10.1016/j.bjane.2024.844517 (PMC11214989; doi:10.1016/j.bjane.2024.844517)
Supplement: Supplementary file 1 [file mmc1.docx]

**BJAN-D-24-00024_Supplementary Material**

**Supporting Information S1 Table** Strengthening the Reporting of Observational Studies in Epidemiology (STROBE).

|  | Item nº | Recommendation | Pages |
| --- | --- | --- | --- |
| **Title and abstract** | 1 | (a) Indicate the study’s design with a commonly used term in the title or the abstract | 1 |
|  |  | (b) Provide in the abstract an informative and balanced summary of what was done and what was found | 2 |
| Introduction | | |  |
| Background/rationale | 2 | Explain the scientific background and rationale for the investigation being reported | 3 |
| Objectives | 3 | State specific objectives, including any prespecified hypotheses | 3 |
| Methods | | |  |
| Study design | 4 | Present key elements of study design early in the paper | 4 |
| Setting | 5 | Describe the setting, locations, and relevant dates, including periods of recruitment, exposure, follow-up, and data collection | 4 |
| Participants | 6 | (a) Give the eligibility criteria, and the sources and methods of selection of participants. Describe methods of follow-up | 4 |
|  |  | (b) For matched studies, give matching criteria and number of exposed and unexposed | ‒ |
| Variables | 7 | Clearly define all outcomes, exposures, predictors, potential confounders, and effect modifiers. Give diagnostic criteria, if applicable | 4 |
| Data sources/ measurement | 8* | For each variable of interest, give sources of data and details of methods of assessment (measurement). Describe comparability of assessment methods if there is more than one group | 4‒5 |
| Bias | 9 | Describe any efforts to address potential sources of bias | 4‒5 |
| Study size | 10 | Explain how the study size was arrived at | 6 |
| Quantitative variables | 11 | Explain how quantitative variables were handled in the analyses. If applicable, describe which groupings were chosen and why | 5 |
| Statistical methods | 12 | (a) Describe all statistical methods, including those used to control for confounding | 5‒6 |
|  |  | (b) Describe any methods used to examine subgroups and interactions | 5‒6 |
|  |  | (c) Explain how missing data were addressed | 5 |
|  |  | (d) If applicable, explain how loss to follow-up was addressed | ‒ |
|  |  | (e) Describe any sensitivity analyses | ‒ |
| Results | | |  |
| Participants | 13* | (a) Report numbers of individuals at each stage of study ‒ e.g., numbers potentially eligible, examined for eligibility, confirmed eligible, included in the study, completing follow-up, and analysed | 6 |
|  |  | (b) Give reasons for non-participation at each stage | 6 |
|  |  | (c) Consider use of a flow diagram | Figure 1 |
| Descriptive data | 14* | (a) Give characteristics of study participants (e.g., demographic, clinical, social) and information on exposures and potential confounders | 6 |
|  |  | (b) Indicate number of participants with missing data for each variable of interest | Table 1 |
|  |  | (c) Summarize follow-up time (e.g., average and total amount) | 6 |
| Outcome data | 15* | Report numbers of outcome events or summary measures over time | 7 |
| Main results | 16 | (a) Give unadjusted estimates and, if applicable, confounder-adjusted estimates and their precision (e.g., 95% Confidence Interval). Make clear which confounders were adjusted for and why they were included | 7 |
|  |  | (b) Report category boundaries when continuous variables were categorized | ‒ |
|  |  | (c) If relevant, consider translating estimates of relative risk into absolute risk for a meaningful time period | ‒ |
| Other analyses | 17 | Report other analyses done ‒ e.g., analyses of subgroups and interactions, and sensitivity analyses | 7 |
| Discussion | | |  |
| Key results | 18 | Summarize key results with reference to study objectives | 8 |
| Limitations | 19 | Discuss limitations of the study, taking into account sources of potential bias or imprecision. Discuss both direction and magnitude of any potential bias | 9 |
| Interpretation | 20 | Give a cautious overall interpretation of results considering objectives, limitations, multiplicity of analyses, results from similar studies, and other relevant evidence | 8‒9 |
| Generalizability | 21 | Discuss the generalizability (external validity) of the study results | 9‒10 |
| Other information | | |  |
| Funding | 22 | Give the source of funding and the role of the funders for the present study and, if applicable, for the original study on which the present article is based | 10 |

**Supporting Information** **S2 Table** Variables included in the Ex-Care model with respective odds ratios and confidence intervals after variables adjustments.

| **Variable** | ***Odds Ratio*** | **95% IC** | **p** |
| --- | --- | --- | --- |
| Age, yr (splines) 17 (ref) | 1.00 | 1.00 |  |
| 30 | 1.09 | 0.55‒2.16 | NS |
| 50 | 1.38 | 0.55‒3.45 | NS |
| 60 | 1.84 | 0.80‒4.26 | NS |
| 70 | 2.70 | 1.11‒6.52 | <0.01 |
| 80 | 3.78 | 1.58‒9.01 | <0.01 |
| 90 | 5.27 | 2.12‒13.11 | <0.01 |
| ASA-PS | 6.66 | 5.65‒7.84 | <0.0001 |
| Major vs non-major | 1.69 | 1.35‒2.13 | <0.0001 |
| Status (non-elective vs. elective) | 4.25 | 3.36‒5.37 | <0.0001 |
| **Mortality Probability ‒ Risk Classes** | | | |
| Class I ‒ <2% | | | |
| Class II – 2%‒5% | | | |
| Class III – 5%‒10% | | | |
| Class IV ‒ >10% | | | |

Adapted from Gutierrez et al., 2021.[8]

**Supporting Information S3 Table** *Checklist* of PACU discharge criteria - all items must be met.

| • Stable vital signs |
| --- |
| • Awake and oriented or with usual sensory pattern |
| • Spontaneous ventilation |
| • Maintains SpO_2_ >90% |
| • Controlled pain |
| • Absence of nausea and vomiting |
| • Absence of bleeding |
| • Absence of motor block or motor block in regression |

**Supporting Information S4 Table** Variables definition.

| **Variable** | **Type** | **Definition** |
| --- | --- | --- |
| Urgent surgery | Categorical | Surgery whose delay in hours implies a risk to life or organ dysfunction. |
| Surgical severity | Categorical | Defined as high, intermediate and low risk according to the Ex-Care model. |
| Bleeding | Categorical | Intraoperative blood loss >500 mL reported in the surgical description and/or medical record. |
| Sepsis | Categorical | Preoperative: Life-threatening organ dysfunction caused by dysfunctional response to infection. Organ dysfunction is characterized by ≥2 points on the SOFA score. |
| Coronary artery disease/Heart failure | Categorical | Preoperative: Previous diagnosis of ischemic heart disease (AMI or angina) or congestive heart failure |
| COPD | Categorical | Preoperative: Previous diagnosis of asthma, reactive airway disease, chronic obstructive pulmonary disease, chronic bronchitis or emphysema |
| Acute kidney injury | Categorical | Preoperative: Reduction in renal function in hours/days classified by AKIN (increase of 0.3 mg.dL^-1^ or increase of 150%‒200% from baseline or diuresis < 0.5 mL.^-1^.kg.h^-1^ for 6-hours) |
| Chronic renal failure | Categorical | Preoperative: Kidney damage and progressive/irreversible loss of kidney function. Rated from stage 2 (eGFR < 90 mL.min^-1^) for treatment purposes according to KDIGO criteria |
| Insulin-dependent DM | Categorical | Preoperative: Diabetes mellitus requiring insulin for glycemic control |
| Anemia | Categorical | Preoperative: Hb < 13 g.dL^-1^ for men; < 12 g.dL^-1^ for women |
| Cancer | Categorical | Preoperative: Malignant neoplasm active in the last 5-years |
| Postoperative vasopressor | Categorical | Postoperative: Use of vasoactive drug for hemodynamic stabilization described in medical records |

SOFA, Sequential [Sepsis-related] Organ Failure Assessment; COPD, Chronic Obstructive Pulmonary Disease; AKIN, Acute Kidney Injury Network; KDIGO, Kidney Disease Improving Global Outcomes; DM, Diabetes Mellitus; eGFR, estimated Glomerular Filtration Rate; Hb, Hemoglobin.
